# Bone Morphogenetic Protein Signaling Restricts Proximodistal Extension of the Ventral Fin Fold

**DOI:** 10.3389/fcell.2020.603306

**Published:** 2020-11-30

**Authors:** Jun Ka, Jun-Dae Kim, Boryeong Pak, Orjin Han, Woosoung Choi, Hwan Kim, Suk-Won Jin

**Affiliations:** ^1^School of Life Sciences, Cell Logistics Research Center, Gwangju Institute of Science and Technology, Gwangju, South Korea; ^2^Yale Cardiovascular Research Center, Section of Cardiovascular Medicine, Department of Internal Medicine, Yale School of Medicine, New Haven, CT, United States; ^3^Center for Cardiovascular Regeneration, Department of Cardiovascular Sciences, Houston Methodist Research Institute, Houston, TX, United States; ^4^Gwangju Institute of Science and Technology, Central Research Facilities, Gwangju, South Korea

**Keywords:** BMP signaling, unpaired fin, ventral fin fold, zebrafish, anisotropic growth

## Abstract

Unpaired fins, which are the most ancient form of locomotory appendages in chordates, had emerged at least 500 million years ago. While it has been suggested that unpaired fins and paired fins share structural similarities, cellular and molecular mechanisms that regulate the outgrowth of the former have not been fully elucidated yet. Using the ventral fin fold in zebrafish as a model, here, we investigate how the outgrowth of the unpaired fin is modulated. We show that Bone Morphogenetic Protein (BMP) signaling restricts extension of the ventral fin fold along the proximodistal axis by modulating diverse aspects of cellular behaviors. We find that lack of BMP signaling, either caused by genetic or chemical manipulation, prolongs the proliferative capacity of epithelial cells and substantially increases the number of cells within the ventral fin fold. In addition, inhibition of BMP signaling attenuates the innate propensity of cell division along the anteroposterior axis and shifts the orientation of cell division toward the proximodistal axis. Moreover, abrogating BMP signaling appears to induce excessive distal migration of cells within the ventral fin fold, and therefore precipitates extension along the proximodistal axis. Taken together, our data suggest that BMP signaling restricts the outgrowth of the ventral fin fold during zebrafish development.

## Introduction

Teleosts are a paraphyletic group composed of diverse species, with characteristic anatomical features that enable living in aquatic environments. Fins, which are the major form of appendages, are arguably the most anatomically distinct features of teleosts. Based on their morphology and number, fins could be divided into two types, unpaired fins and paired fins (Freitas et al., 2014; Larouche et al., 2017). Recent phylogenetic analyses indicate that unpaired fins are more ancient structures, evolved in ostracoderms approximately 100 million years earlier than the advent of paired fins (Coates, 1995; Shubin, 1995). Therefore, these two types of fins may have emerged by separate evolutionary events (Coates, 1995; Grandel and Schulte-Merker, 1998, van den Boogaart et al., 2012). Consistent with this idea, there are a number of key differences between unpaired and paired fins. For instance, unpaired fins are medially located, while paired fins are located in the ventrolateral region of the body with pectoral fins and pelvic fins respectively at the anterior and posterior extremity of the trunk. During development, unpaired and paired fins are derived from distinct structures; while unpaired fins arise from the paraxial mesoderm, paired fins diverge from the lateral plate mesoderm. In addition, paired fins are connected to the body by girdles which allow articulation, while unpaired fins are extended directly from the body wall as a result of mesenchymal expansion within embryonic fin folds (Grandel and Schulte-Merker, 1998; Yamanoue et al., 2010).

Since paired fins appear to have more resemblance to tetrapod appendages than unpaired fins in terms of morphology and function (Mercader, 2007), the development of paired fins in teleosts has been extensively investigated and underlying cellular and molecular mechanisms have been identified. For instance, it has been shown that Sonic Hedgehog (SHH) signaling, which is a key factor governing the patterning of tetrapod appendages, also appears to be instrumental for the formation of the pectoral fins in teleosts (Hadzhiev et al., 2007). In addition, a number of transcription factors that are essential for development of the appendages in tetrapods such as *hand2* and *tbx5* similarly regulates development of the pectoral fins (Mercader, 2007). Recently, Bone morphogenetic protein (Bmp) signaling, which provides diverse function during development including specification of the embryonic axis (Graff, 1997), has been shown to maintain the stereotypic morphology of the pectoral fins by modulating gradient scaling within developing pectoral fins (Mateus et al., 2020).

Compared to paired fins, cellular and molecular mechanisms regulating the development of unpaired fins remain largely unknown. During development, unpaired fins are derived from the fin folds which are continuous folds of the epidermal tissue. In zebrafish, the median fin fold, which is an extension of the surface epidermis to the dorsal and caudal surface of the embryo posterior to the 8th somite, appears at approximately 22 h post-fertilization (hpf). Subsequently, the dorsal fin fold generates the dorsal fin, while the ventral fin fold is further divided into the anal and caudal fin (van den Boogaart et al., 2012). Based on distinct evolutionary history, it has been suggested that developing unpaired fins and paired fins are likely to differently respond to the identical signaling input. For instance, Shh signaling, which is the key factor modulating the development of paired fins, only exerts indirect effects on the development of unpaired fins (Hadzhiev et al., 2007).

In this report, we investigated the role of Bmp signaling on the morphogenesis of unpaired fin, using ventral fin fold in zebrafish embryo as a model. We find that Bmp signaling negatively modulates the outgrowth of the ventral fin fold. We show that inhibition of Bmp signaling at late somitogenesis stages significantly extends the ventral fin fold along the proximodistal axis without affecting other organs. We demonstrate that Bmp signaling appears to restrict the proximodistal growth of unpaired fin by limiting proliferative capacity and thereby promotes quiescence of epithelial cells. In addition, we show that Bmp signaling modulates the orientation of cell division in the ventral fin fold, and thereby regulates innate anisotropic growth of unpaired fin. Taken together, our data suggest that Bmp signaling could negatively modulate the morphogenesis of unpaired fins in zebrafish.

## Materials and Methods

### Zebrafish Husbandry

All zebrafish (*Danio rerio*) were maintained under standard conditions in accordance with institutional and national guidelines, approved by the Institutional Animal Care and Use Committee. Fish stocks were maintained in an animal facility at 28.5°C on a 14 h/10 h light/dark cycle. The wild-type strain used was AB. Published strains used in this study include *Tg(fli1a:EGFP)* (Lawson and Weinstein, 2002). *Tg(-3.5ubb:Cerulean-gmnn-2A-mCherry-cdt1)* (Bouldin and Kimelman, 2014).

### Chemical Treatment and Morpholino Injection

DMH1 (Sigma-Aldrich, St. Louis, MI, United States, D8946) used in experiments was reconstituted at stock concentrations (10 mM) in DMSO solvent. Embryos were dechorionated by hand using forceps and dechorionated embryos were transferred to 6-well plates. Embryo media with appropriate drug concentration or vehicle control was added to each well. For DMH1 treatments, embryos were treated from 28 hpf to 76 hpf. Embryos were incubated at 28.5°C for the duration of the drug treatment. For phenotypic analysis, embryos were mounted in 2% methylcellulose and documented using Leica M165FC microscope and Leica MC 170 HD camera. *P* values were calculated using unpaired t-test with Welch’s correction (GraphPad Prism7).

The sequence of *alk3a*, *alk3b*, *alk6a*, and *alk6b* morpholinos were used as previously described (Little and Mullins, 2009; Neumann et al., 2011) (Supplementary Table 1). Each morpholino was injected into 1-cell stage wild-type zebrafish embryos. The morpholino doses used are 1, 2, 4, 8, 16, and 32 ng. Embryos were collected at 52 and 76 hpf and mounted in 2% methylcellulose and documented using Leica M165FC microscope and Leica MC 170 HD camera.

### Whole Mount *in situ* Hybridization

RNA probes were synthesized using DIG RNA Labeling Kit (Roche) per manufacturer’s instruction. Fragments for synthesizing probes were amplified from wild type cDNA and cloned into pGEM-T Easy by TA cloning (Promega). The sequence of primers for PCR amplification are the shown in the Supplementary Table 1. Probes were synthesized using T7 and SP6 promoters. Wild-type embryos of the appropriate stage were fixed in 4% PFA at 4°C for 24 h. Following fixation, embryos were dehydrated in methanol and stored at −20°C. Whole mount *in situ* hybridization was performed as follows. Hybridized probes were detected using anti-digoxigenin (DIG) antibodies tagged with alkaline-phosphatase (AP) (Roche) using NBT/BCIP (Roche) solution per manufacturer’s instructions. Stained embryos were mounted in 90% glycerol/PBST and imaged using a Leica M165FC microscope and Leica MC 170 HD camera.

### EdU Incorporation and Immunofluorescence

Embryos were dechorionated at the desired stage and treated with the appropriate concentration of drug. At 48 hpf stage, EdU (Invitrogen, Carlsbad, CA, United States) (2 mM, 2 nl volume) solution was injected into the yolk of each embryo. Injected embryos were incubated at 28.5°C for 5 h. Embryos were washed thoroughly in fresh embryo media and fixed in 4% PFA at 4°C for 24 h. Following fixation, embryos were permeabilized by washing with 1% Tween-20 in PBS for 30 min. The EdU reaction mix was prepared per manufacturer’s instructions. Embryos were then transferred to the reaction mix and incubated in the dark for one hour at room temperature. For imaging, embryos were washed in PBST and mounted in 1% low melt agarose and imaged using a Zeiss LSM 700 confocal microscope. *P* values were determined using unpaired t-test with Welch’s correction (GraphPad Prism7).

Embryos were fixed after drug treatment in 4% PFA for 24 h at 4°C. Embryos were permeabilized by washing with 1% Tween-20 in PBS for 30 min. Whole mount immunofluorescence was performed as previously described (Kim et al., 2012). pSMAD-1/5 rabbit monoclonal primary antibody (1:100 dilution) (Cell Signal Technology, Danvers, MA, United States, 41D10) was used. AlexaFluor-488 (Invitrogen, Carlsbad, CA, United States) and AlexaFluor-555 (Invitrogen) conjugated secondary antibodies were used to detect primary antibody signaling. Nuclei were counterstained with TO-PRO-3 (Invitrogen, Carlsbad, CA, United States). For F-actin staining, AlexaFluor-568 conjugated phalloidin (Invitrogen, Carlsbad, CA, United States) was used. Fluorescent images were taken using a Zeiss LSM 700 confocal microscope and a Zeiss Lightsheet Z.1.

### Live Imaging of Zebrafish Embryos

To assess the behavioral changes of cells within the ventral fin fold in response to BMP signaling, 48 hpf *Tg(-3.5ubb:Cerulean-gmnn-2A-mCherry-cdt1)* embryos were treated with 2 μM DMH1. Fish were anesthetized in tricaine in embryo media and mounted in 1% low melt agarose in embryo media with tricaine for imaging. To prevent shrinkage, confocal dish was filled with embryo media with DMH1 and tricaine. Fluorescent images were captured every 15 min using a Zeiss LSM 700 confocal microscope from 48 hpf to 60 hpf. Images were processed using Zen and IMARIS software. Dividing cells were counted and analyses were performed using IMARIS and GraphPad Prism 7 and Origin.

### Image Analyses

To count the number of dividing cells, nuclei undergoing karyokinesis were counted. The coordinates of nuclei of original cells were measured and relative position was calculated. The underneath of caudal vein was assigned as relative position 0 and the distal end line of ventral fin fold was assigned as relative position 100. Distribution and density was defined by Kernel density smoothing. Significance were determined using Mann-Whitney test (Origin). To quantify the orientation of cell division, the coordinates of nuclei of daughter cells were measured and degree of orientation of dividing cells were calculated using Microsoft Excel. The value of calculated degree was presented in polar contour plot in both directions.

To investigate migratory behavior of individual cells in ventral fin fold, 2D-projected time-lapse images from 50 hpf to 60 hpf were analyzed using IMARIS. Center of individual cells in ROI (region of interest) were automatically spotted and subsequently manually corrected. We filtered out low-quality cells following the criteria such as estimated XY diameter and spot intensity quality. Cells which are not considered epithelial cells such as clodronate-sensitive immune cells and PTU-sensitive melanophores were excluded. The cells which were tracked across the border line of ROI were excluded. Spots were automatically tracked using Autoregressive motion tracking algorithm. As a threshold to sort reliable tracks, we used track duration above 21 frames and max distance less than 2 μm between each frame. Tracks were presented in scatter plot by gathering the origin of tracks. The coordinates of spots per each frame from 1 to 40 were presented in contour plot.

## Results

### Bmp Signaling Limits Outgrowth of the Ventral Fin Fold

**FIGURE 1:**
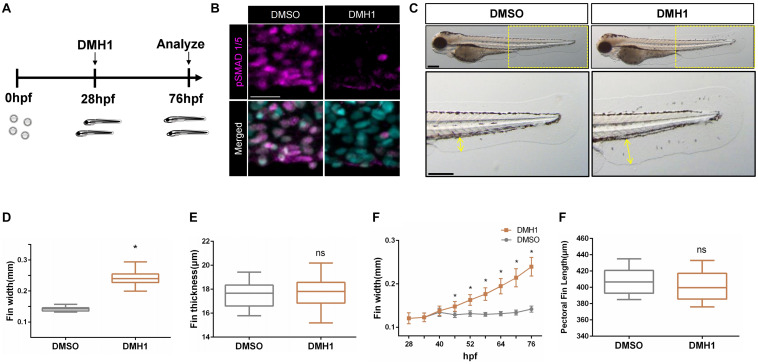
Bmp signaling limits the distal extension of the ventral fin fold in zebrafish. **(A)** Schematic illustration of experiments. Zebrafish embryos were treated with DMH1 at 28 hpf, and the effects of Bmp inhibition were assessed at 76 hpf. **(B)** Immunohistochemistry of pSMAD1/5 in DMSO- or DMH1-treated embryos within the ventral fin fold. Scale bar: 20 μm. **(C)** Brightfield images of 72 hpf DMSO- and DMH1-treated embryos. Yellow boxes in the top panels are shown in detail. Scale bar: 200 μm. **(D)** Quantification of the length of the ventral fin fold. The length of the yellow lines in **(C)** was quantified (*n* = 53). **(E)** Quantification of the width of the ventral fin fold (*n* = 11). **(F)** Cumulative growth of the ventral fin fold between 28 hpf to 76 hpf. The proximodistal extension of the ventral fin fold continues in DMH1-treated embryos. **(G)** Quantification of the length of the pectoral fins (*n* = 11). *p < 0.0001. ns: not significant.

Previously, Bmp signaling has been shown to modulate the outgrowth of paired fins (Mateus et al., 2020). However, the mechanisms whereby Bmp signaling modulates morphogenesis of unpaired fins, which have evolutionarily and developmentally distinct origin from paired fins (Larouche et al., 2017), remains largely unknown. We postulated that unpaired fins may respond differently to Bmp signaling as shown in paired fins. To further examine this idea, we analyzed the effects of Bmp inhibition on the ventral fin fold by treating zebrafish embryos with DMH1, a selective chemical antagonist against Bmp signaling (Hao et al., 2010), from 28 hpf to 76 hpf (Figure 1A). Treatment with 2 μM DMH1 effectively abrogated the deposition of phosphorylated Smad1/5 in the ventral fin fold (Figure 1B). In DMH1-treated embryos, the distance from the base to the edge of the ventral fin fold was substantially increased in a dose-dependent manner, without affecting the thickness of the ventral fin fold (Figures 1C–E and Supplementary Figure 1A). In DMH1-treated embryos, the ventral fin fold continued to extend along the proximodistal axis even after the stage when the ventral fin fold ceased to grow in DMSO-treated embryos (Figure 1F).

To further define the critical period when Bmp signaling limits growth of the ventral fin fold, embryos were treated with DMH1 for 6 h, starting from 28 hpf, and the effects of Bmp inhibition on the outgrowth of the ventral fin fold was assessed at 76 hpf. We found that the ventral fin fold is susceptible to inhibition of Bmp signaling between 28 hpf and 52 hpf. In particular, the extension of the ventral fin fold along the proximodistal axis was most pronounced in embryos treated with DMH1 between 28 hpf and 34 hpf (Supplementary Figure 1B). The effects of Bmp inhibition appeared to be specific to the ventral fin fold, since the morphology of other organs remained unaltered including the pectoral fins, with a notable exception of the melanophores (Figure 1G and Supplementary Figures 2A–G). The number of melanophores, which has shown to be negatively regulated by Bmp signaling (Gramann et al., 2019), was concomitantly increased in DMH1-treated embryos (Supplementary Figures 2D,E). Taken together, our data suggest that Bmp signaling selectively restricts the extension of the ventral fin fold along the proximodistal axis in a stage-dependent manner. It is interesting to note that we did not find any morphological effects of DMH1 treatment on the pectoral fins, of which outgrowth has been recently reported to be regulated by Bmp signaling (Mateus et al., 2020) (Supplementary Figure 2H, Movie 1). Since the development of the ventral fin fold occurs much earlier than the pectoral fins, it is fathomable that spatiotemporal requirement of Bmp signaling on the outgrowth of unpaired fins and paired fins may be distinct. Consistent with this idea, we found that the caudal fin, an another unpaired fin which develop adjacent to the ventral fin fold at the same developmental period, was similarly extended along the anteroposterior axis in the absence of Bmp signaling (Supplementary Figures 2H,I).

To determine how Bmp signaling modulates outgrowth of unpaired fins along the proximodistal axis, we first sought to identify receptors which mediate Bmp signaling in the ventral fin fold. Among Bmpr1, *alk3a*, *alk3b, alk6a*, and *alk6b* appear to be highly expressed within the ventral fin fold at 28 hpf (Supplementary Figure 3A). To further elucidate whether these receptors mediate Bmp signaling within the ventral fin fold, embryos were injected with Morpholinos (MOs) against *alk3a*, *alk3b, alk6a*, or *alk6b* and the resulting phenotypes were assessed. Inhibition of individual Bmpr1, however, did not recapitulate the phenotype of the ventral fin fold in DMH1-treated embryos (Supplementary Figure 3B). Conversely, ectopic expression of dominant-negative Bmpr1 (DN-Bmpr1) (Pyati et al., 2005) led to proximodistal extension of the ventral fin fold, suggesting that multiple Bmpr1s may synergistically transduce Bmp signaling within the ventral fin fold to limit the proximodistal extension (Supplementary Figure 3C).

We then sought to identify how Bmp signaling could modulate the outgrowth of the ventral fin fold. Since it has been previously reported that BMP signaling could attenuate Epidermal Growth Factor (EGF) signaling pathways in a number of cell types (Scholze et al., 2014), we examined whether Bmp signaling similarly interferes with Egf signaling during the outgrowth of the ventral fin fold. In DMH1-treated embryos, expression of Egf signaling components was significantly increased (Supplementary Figure 3D), hinting that Bmp signaling may modulate the outgrowth of ventral fin fold by negatively regulating the expression of Egf signaling components.

### Bmp Signaling Limits Proliferative Capacity Within the Ventral Fin Fold

**FIGURE 2:**
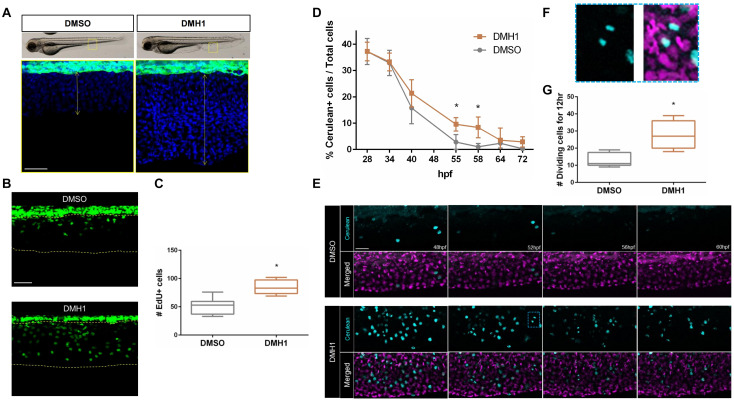
Bmp signaling regulates proliferative capacity of cells in the ventral fin fold. **(A)** The number of cells in the ventral fin fold of DMSO- or DMH1-treated embryos. Nuclei were visualized by TOPRO staining. The areas within the rectangles in the top panels are shown in high magnification. Scale bar: 50 μm. **(B)** EdU labeling of proliferating cells in DMSO- or DMH1-treated embryos (*n* = 9). Scale bar: 50 μm. **(C)** Quantification of EdU positive cells in the ventral fin fold of DMSO- or DMH1-treated embryos (*n* = 9). **(D)** Quantification of Cerulean positive cells in the ventral fin fold of DMSO- or DMH1-treated embryos at different developmental stages. **(E)** Frames taken at 48, 52, 56, and 60 hpf from time-lapse images of DMSO- or DMH1-treated embryos with Dual FUCCI background between 48 hpf to 60 hpf. DMH1-treated embryos retain significantly increased number of proliferating cells. The areas within the blue rectangle are shown in **(F)** in high magnification. Scale bar: 50 μm. **(F)** Example of the dividing cells within the ventral fin fold. **(G)** Quantification of the number of proliferating cells in the ventral fin fold (*n* = 5). *p < 0.0001.

Next, we assessed the total number of cells within the ventral fin fold in DMSO- or DMH1-treated embryos. Attenuation of Bmp signaling by DMH1 treatment significantly increased the number of cells (Figure 2A). Accordingly, DMH1-treated embryos possessed substantially increased numbers of EdU^+^ cells compared to DMSO-treated embryos, suggesting that increased cell proliferation may promote the extension of the ventral fin fold along the proximodistal axis in the absence of BMP signaling activity (Figures 2B,C).

Consistent with this idea, DMH1-treated *Tg(-3.5ubb:Cerulean-gmnn-2A-mCherry-cdt1)* embryos (henceforth Dual FUCCI) (Bouldin and Kimelman, 2014), which labels cells at G1 stage with mCherry and those at S, M, and G2 stage with Cerulean, appeared to have a significantly increased number of proliferating cells compared to DMSO-treated embryos. At 28 hpf, the number of Cerulean^+^ cells in the ventral fin fold was comparable in DMSO- and DMH1-treated embryos (Figure 2D and Supplementary Figure 4). While the Cerulean^+^ cells in the ventral fin fold persisted in DMH1-treated embryos until 72 hpf, the number of the Cerulean^+^ cells in DMSO-treated embryos gradually diminished over time and by 55hpf, cells within the ventral fin fold became predominantly quiescent, containing only mCherry^+^ cells (Figure 2D and Supplementary Figure 5). Considering that the number of the Cerulean^+^ cells in DMH1-treated and DMSO-treated control embryos were comparable at 28 hpf, it does not appear that inhibition of Bmp signaling increases the proliferation rate of cells within the ventral fin fold at early stages, but sustains their proliferative capacity.

To better assess the dynamics of cell proliferation in the ventral fin fold in the absence of Bmp signaling, we examined the outgrowth of the ventral fin fold in detail by performing time-lapse imaging on the DMH1- or DMSO-treated Dual FUCCI transgenic embryos between 48 hpf and 60 hpf (Figure 2E and Supplementary Movies 2, 3). Consistent with our previous observations, the number of cells undergoing karyokinesis was significantly increased in DMH1-treated embryos (Figures 2F,G). Taken together, our data suggest that BMP signaling is likely to promote quiescence of cells within the ventral fin fold, and thereby limiting the size of the ventral fin fold.

### Bmp Signaling Regulates Cell Behaviors During Proliferation in the Ventral Fin Fold

**FIGURE 3:**
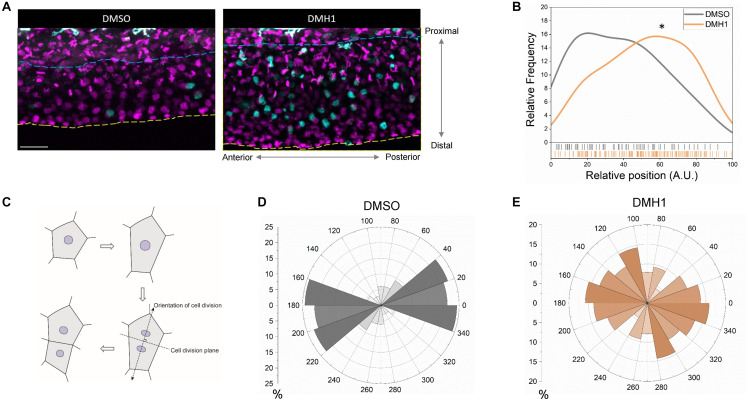
Bmp signaling modulates orientation of cell division in the ventral fin fold. **(A)** Time -lapse image showing cells undergoing karyokinesis in the ventral fin fold in DMSO- or DMH1-treated embryos along the proximodistal axis. The blue dashed lines and the yellow dashed line denote the most proximal end (relative position 0 along the proximodistal axis) and the most distal end (relative position 100 along the proximodistal axis) in the ventral fin fold. Scale bar: 50 μm. **(B)** Quantification of the proliferative capacity in the ventral fin fold of DMSO- or DMH1-treated embryos along the proximodistal (*n* = 5 for DMSO-treated, and 5 for DMH1-treated). ^∗^*p* < 0.001. **(C)** Schematic illustration on measuring the orientation of cell division. **(D,E)** Quantification of the orientation of cell division in DMSO- **(D)** or DMH1- **(E)** treated embryos (*n* = 5 for DMSO-treated, and 5 for DMH1-treated).

Since it is possible that Bmp signaling can selectively enhance the proliferative capacity of cells within a specific sub-region within the ventral fin fold to modulate the outgrowth, we examined the distribution of proliferating cells along the proximodistal axis. Within the ventral fin fold, we identified a number of cell types with distinct morphology. While the majority of the cells appear to be have stereotypic morphology of epithelial cells, there were two types of cells with distinct morphology; highly mobile cells and highly arborized cells. Since clodronate liposome injection, which abrogates macrophages (Danenberg et al., 2002), abrogated the majority of highly mobile cells. In addition, phenylthiourea treatment, which induces selective apoptosis of melanophores in zebrafish (McNeill et al., 2007), drastically reduced the number of highly arborized cells (Supplementary Figure 4D). Therefore, these cells do not appear to be epithelial cells in nature, and were excluded from further analyses. Quantification on the relative positions of the proliferating cells along the proximodistal axis revealed that DMSO- and DMH1-treated embryos have distinct pattern (Figures 3A,B). While proliferating cells in DMSO-treated embryos were largely confined to the more proximal region of the ventral fin fold, in DMH1-treated embryos, proliferating cells were more evenly distributed (Figure 3B). Therefore, it appears that excessive proliferation, in particular at the distal region of the ventral fin fold, serves as an important contributing factor for unregulated outgrowth of the ventral fin fold along the proximodistal axis in the absence of Bmp signaling.

Since the orientation of cell division could modulate the direction of outgrowth, we examined whether the orientation of cell division was different between DMSO- and DMH1-treated embryos (schematic diagram is shown in Figure 3C). In DMSO-treated embryos, the orientation of cell division generally favored elongation along the anteroposterior axis rather than the proximodistal axis, with the majority of cell division, occurred between -20 to 40 degree (Figure 3D), hinting that the ventral fin fold undergoes anisotropic growth along the anteroposterior axis. However, in DMH1-treated embryos, the orientation of cell division appeared to be shifted between 100 to 140 degrees, preferentially depositing newly formed cells along the proximodistal axis (Figure 3E). Therefore, inhibition of Bmp signaling not only delimits the proliferative capacity of cells within the ventral fin fold, it also alters the innate anisotropic growth of the ventral fin fold by facilitating the deposition of newly formed cells along the proximodistal axis.

### Bmp Signaling Limits Migratory Behaviors of Cells Within the Ventral Fin Fold

**FIGURE 4:**
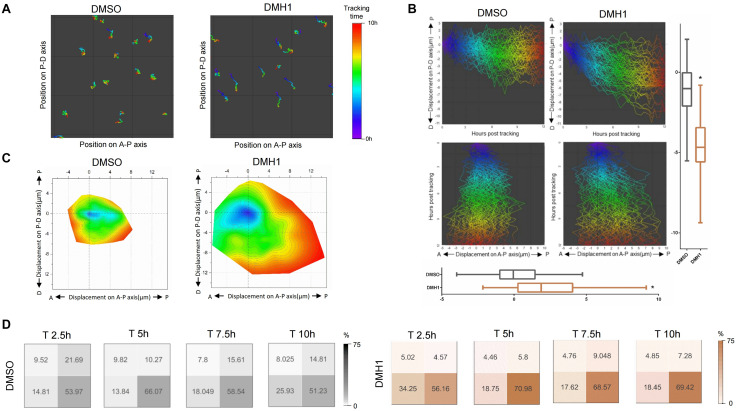
Bmp signaling does not facilitate distal migration of cells in the ventral fin fold. **(A)** Migratory tract of cells within the ventral fin fold in DMSO- or DMH1-treated embryos between 50 hpf to 60 hpf. Migratory tract was visualized with anteroposterior and proximodistal axes as X and Y axes respectively. Position of individual cells within the ventral fin fold is color-coded. Areas within the sample #1 and sample #4 in the Supplementary Figure 5 are shown. **(B)** Composite images depicting migratory tract of cells within the ventral fin fold in DMSO- or DMH1-treated embryos between 50 hpf to 60 hpf embryos. Migratory tract analyzed in sample #1 and sample E4 in the Supplementary Figure 6 are shown. Collective migratory tract was visualized with anteroposterior and proximodistal axes as X and Y axes respectively. Cells within the ventral fin fold of DMH1-treated embryos tend to migrate more to the distal and posterior ends (*p* < 0.0001). **(C)** Contour map of cell migratory tract of cells within the ventral fin fold of DMSO- or DMH1-treated embryos between 50 hpf to 60 hpf. Position of individual cells over time are shown with anteroposterior and proximodistal axes as X and Y axes respectively. While cells within the ventral fin fold of DMH1-treated embryos expanded toward the distal and posterior ends compared to those of DMSO-treated embryos. Quantification of the total migration distance in DMSO- (*n* = 3) or DMH1-treated embryos (*n* = 3). **(D)** Position of individual cells within the ventral fin fold in DMSO- (*n* = 3) or DMH1-treated embryos (*n* = 3) at distinct developmental stages. In DMH1-treated embryos, the frequency of migrating cells localized within the proximal quadrants was increased. Analyses were performed on 50 hpf zebrafish embryos for ten hours.

To assess whether directed cell migration also contributes to the proximodistal extension of the ventral fin fold in DMH1-treated embryos, we analyzed migratory behaviors of individual cells within the ventral find fold, in particular, those localized within the mid-section of the ventral fin fold in respect to the proximodistal axis (Figure 4A and Supplementary Figure 5). To quantify the cumulative migration distance, migratory paths of individual mCherry positive cells were overlaid, and migratory path as well as their position within the ventral fin fold upon completion of migration were assessed (Figure 4B and Supplementary Figure 6). Compared to cells in the ventral fin fold of DMSO-treated embryos, those of DMH1-treated embryos showed significantly increased migration distance (Figure 4B). Within 10 h window, cells within the ventral fin fold of DMSO-treated embryos migrated an average of 1.11 μM along the proximodistal axis from their initial positions. In comparison, those of DMH1-treated embryos migrated an average of 4.61 μM from their initial positions along the proximodistal axis. Similarly, cells within the ventral fin fold of DMSO- or DMH1-treated embryos migrated an average of 0.03 and 2.38 μM along the anteroposterior axis, respectively, indicating that cells displayed the propensity to migrate further toward the posterior and distal end of the ventral fin fold in the absence of Bmp signaling (Figure 4B).

To better determine how Bmp signaling affects the direction of migration, contour maps based on the migratory tract of individual cells were constructed in DMSO- or DMH1-treated embryos (Figure 4C). We found that cells in the ventral fin fold possessed the innate propensity to migrate toward the distal and posterior ends; while the general shape of the contour maps was comparable in both DMSO- and DMH1-treated embryos with similar directionality, cells in DMH1-treated embryos appeared to migrate further, in particular, toward the distal end of the ventral fin fold (Figure 4C). To assess the preference in the direction of migration, we divided the cells within the area of interest into four quadrants, and calculated the frequency of cells localized within each quadrant. While cells in both DMSO- and DMH1-treated embryos showed similar preference to migrate toward the distal end of the ventral fin fold early on, this tendency was augmented in DMH1-treated embryos at later stage, and the predominant portion of cells became localized to the distal quadrants (Figure 4D). Therefore, Bmp signaling appears to prevent excessive navigating behaviors of cells and is likely to restrict the distal migration of the cells within the ventral fin fold. Taken together, our data suggest that Bmp signaling limits the proliferative capacity, maintains the orientation of cell division, and negatively regulates the distal migration within the ventral fin fold, which collectively help to maintain the innate anisotropic growth of the ventral fin fold along the anteroposterior axis.

## Discussion

In this report, we demonstrate that Bmp signaling provides indispensable inputs to modulate diverse aspects of ventral fin fold morphogenesis. We show that Bmp signaling promotes quiescence by limiting the proliferative capacity of cells within the ventral fin fold. In addition, Bmp signaling maintains the innate tendency of the ventral fin fold to grow along the anteroposterior axis by dictating the orientation of cell division. Taken together, our data show that Bmp signaling negatively regulates outgrowth of the ventral fin fold by limiting the extension of developing ventral fin fold along the proximodistal axis.

While Bmp signaling promotes outgrowth of the pectoral fins (Mateus et al., 2020), it appears to restrict outgrowth of the ventral fin fold. Two mutually non-exclusive scenarios could explain how Bmp signaling could differently modulate the outgrowth of the ventral fin fold and pectoral fins. First, it is possible that the effects of Bmp signaling on the cells within the pectoral fins and the ventral fin fold could be distinct due to the intrinsic differences of signaling receiving cells, in line with the idea that Bmp signaling could generate a context-depend outcomes (Kim et al., 2014). Therefore, it is conceivable that Bmp signaling could be interpreted as a positive cue for proliferation and migration by the cells within the pectoral fins, while could be perceived as a negative by the cells within the ventral fin fold. Alternatively, it is possible that Bmp signaling could elicit opposite outcomes in a stage-dependent manner. Since the time window whereby Bmp signaling modulates the outgrowth of these two fin structures is clearly distinct, it is possible that Bmp signaling could function to inhibit excessive outgrowth of the fin structure during early developmental stages, while could promote outgrowth of the fin structure at later stages, similar to its effects on cardiac differentiation (de Pater et al., 2012). Considering that paired fins have emerged much later than unpaired fins during vertebrate evolution (Freita et al., 2017), we speculate that Bmp signaling may have adopted a novel role of promoting directional outgrowth of paired appendages upon the advent of paired fins.

Previously, it has been proposed that localized cell proliferation within the distal region, particularly in response to the signals emanating from the distal tip, serves as a major driving force for the proximodistal extension of the tetrapod appendages (Boehm et al., 2010). For instance, the apical ectodermal ridge (AER), a specialized structure at the distal tip of limb buds, provides a plethora of morphogens to induce spatially restricted cell proliferation in the distal region of the appendages to allow elongation of the developing limbs (Johnson and Tabin, 1997; Zuniga, 2015). In the ventral fin fold, however, we did not find any evidence indicating a spatial distribution of proliferating cells within the ventral fin fold. On the contrary, we found that actively proliferating cells are evenly distributed within the ventral fin fold at early stages. Based on our data, we propose that temporal regulation of cell proliferation is likely to be more critical than spatial regulation in the ventral fin fold, and potentially in other unpaired appendages. We speculate that the spatially uniformed yet temporally restricted proliferation may allow rapid growth of unpaired fins and potentially increase the overall fitness of the individual by providing locomotive capacity at early developmental stages.

Despite the distinct modes of regulation on cell proliferation, the ventral fin fold in zebrafish appears to undergo stereotypic extension based on anisotropic growth, as previously reported in paired fins (Mateus et al., 2020). We found that the ventral fin fold tends to extend along the anteroposterior axis, hinting that the ventral fin fold may undergo anisotropic growth during development. Since we were not able to identify any evidence supporting localized cell proliferation within the ventral fin fold which could prime anisotropic growth of developing ventral fin fold, it is likely that alternative modes may be used to enable anisotropic growth of the ventral fin fold. Consistent with this idea, we found that dividing cells within the ventral fin fold of DMSO-treated embryos have an innate propensity of aligning cell division along the anteroposterior axis. Inhibition of Bmp signaling appears to alter the orientation of cell division along the proximodistal axis, therefore, distally extends the ventral fin fold.

In tetrapod limb buds, BMP7, which binds to ALK3 and ALK6, appears to be the most abundant BMP ligand (Robert, 2007). Similarly, in zebrafish, Bmp7 is highly expressed in both paired and unpaired fins (Shawi and Serluca, 2008). However, our MO analyses did not support the idea that the cognate receptors for Bmp7, Alk3a/b, and Alk6a/b are important for Bmp signaling-mediated regulation of the ventral fin fold outgrowth. Rather, it appears that multiple BMP receptors collectively mediate Bmp signaling within the ventral fin fold. Considering that BMP receptors can heterodimerize to transduce BMP signaling in a context-dependent manner (Hartung et al., 2006), and the receptor-ligand pairing of BMP signaling is known to be promiscuous (Antebi et al., 2017), it is tempting to speculate that an atypical ligand-receptor complex may regulate ventral fin fold outgrowth. Our observation that DN-Bmpr1 could recapitulate the DMH1-induced phenotype of the ventral fin fold supports this notion.

While our data collectively suggest that Bmp signaling modulates outgrowth of unpaired fin, there are a number of limitations which would warrant further investigation on the role of Bmp signaling on ventral fin fold; our analyses were primarily based on the loss-of-function analyses using chemical antagonists at embryonic stages, therefore, it is possible that our analyses might have not been able to capture dynamic changes in the effects of Bmp signaling on the ventral fin fold and its descendants during post-embryonic stages. It would be interesting to examine whether Bmp signaling exerts similar effects on the maintenance and regeneration of the adult fins. However, manipulating Bmp signaling to examine the effects on fin regeneration and/or maintenance may not be technically feasible given that inhibition of Bmp signaling during regeneration could interfere with angiogenesis, which is essential for regeneration and maintenance. In addition, it is not clear whether ectopic activation of Bmp signaling could attenuate the outgrowth of the ventral fin fold. Further analyses using transgenic lines which allow a tissue specific over-expression of Bmp signaling would be needed to precisely delineate the spatiotemporal requirement of Bmp signaling for the outgrowth of the fins. Furthermore, it is unclear how Bmp signaling exerts its effects on outgrowth of the ventral fin fold at molecular level, while our preliminary analyses alluded that Bmp signaling may attenuate Egf signaling by negatively regulating the transcription of key receptors. Therefore, further analyses would help us to fully appreciate the complexity of cellular and molecular mechanisms whereby Bmp signaling modulates the outgrowth of unpaired fins.

Our data suggest that Bmp signaling negatively modulates cell proliferation in the ventral fin fold. While Bmp signaling is generally considered to promote cell proliferation during development, it has been shown to adversely affect cell proliferation in certain scenarios. For instance, it has been shown that Bmp signaling negatively modulates the size of hair follicles by inhibiting cell cycle-related genes (Plikus et al., 2008). In addition, BMP signaling has been shown to inhibit cell proliferation of *Drosophila* anterior and posterior midgut (Guo et al., 2013), and human gastric carcinoma cells (Lee et al., 2016; Sun et al., 2020). Similarly, in regeneration, BMP signaling appears to negatively modulate the proliferation of epithelial cells (Mou et al., 2016; Tadokoro et al., 2016, Qi et al., 2017). Considering that the majority of tissues of which proliferation is negatively modulated by BMP signaling appear to be epidermal in origin, and the dominant cell type within the developing ventral fin fold is the epidermis, it is plausible that the role of BMP signaling in maintaining homeostasis of epidermal tissue is conserved among distinct phyla, representing an ancient function of BMP signaling during development. Considering that the regulation of cell proliferation is critical for regeneration as well as the pathogenesis of diverse human diseases, better understanding of cell type-dependent regulation of BMP signaling on cell proliferation could provide theoretical framework to manipulate cell proliferation in clinical settings.

## Data Availability Statement

The original contributions presented in the study are included in the article/Supplementary Material, further inquiries can be directed to the corresponding author/s.

## Ethics Statement

The animal study was reviewed and approved by Gwangju Institute of Science and Technology.

## Author Contributions

JK, J-DK, BP, WC, OH, and HK performed the experiments. JK, J-DK, BP, WC, and HK analyzed the data. J-DK and S-WJ guided the experiments. S-WJ wrote and all authors edited the manuscript.

## Conflict of Interest

The authors declare that the research was conducted in the absence of any commercial or financial relationships that could be construed as a potential conflict of interest.
