# Supplementary material for: Bone Morphogenetic Protein Signaling Restricts Proximodistal Extension of the Ventral Fin Fold
Source: Front Cell Dev Biol. 2020 Nov 30;8:603306. doi: 10.3389/fcell.2020.603306 (PMC7734333; doi:10.3389/fcell.2020.603306)
Supplement: Supplementary file 1 [file Data_Sheet_1.DOCX]

**SUPPLEMENTARY MATERIALS**

**Supplementary Figures**

**
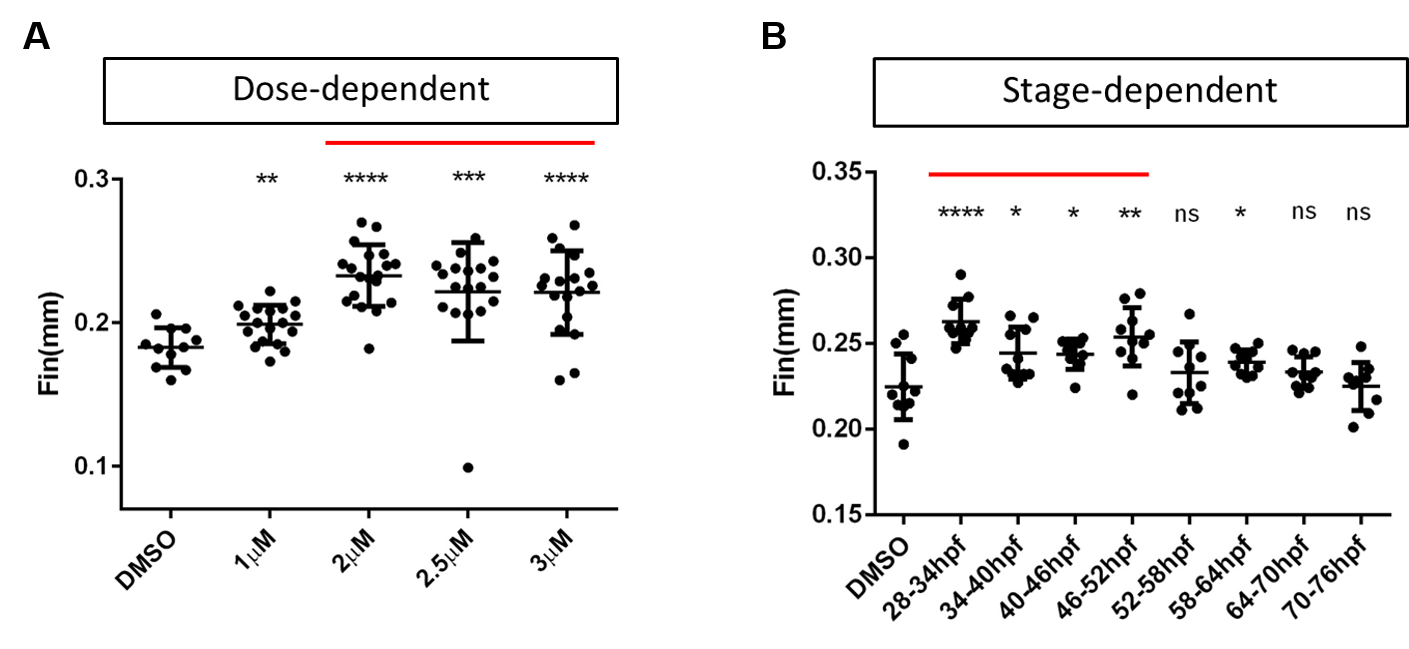
**

**Supplementary Figure 1: Inhibition of Bmp signaling promotes the outgrowth of the ventral fin fold in a dose-dependent and a stage-dependent manner**

(**A**) Embryos were treated with different doses of DMH1 at 28hpf and the distance from the base to the distal end of the ventral fin fold was measured at 76hpf. (**B**) The proximodistal extension of the ventral fin fold in embryos treated with DMH1 at different developmental stages was measured at 76hpf to identify the sensitive period of BMP signaling inhibition. **p*<0.05, ***p*<0.01, ****p*<0.001, *****p*<0.0001. ns: not significant.


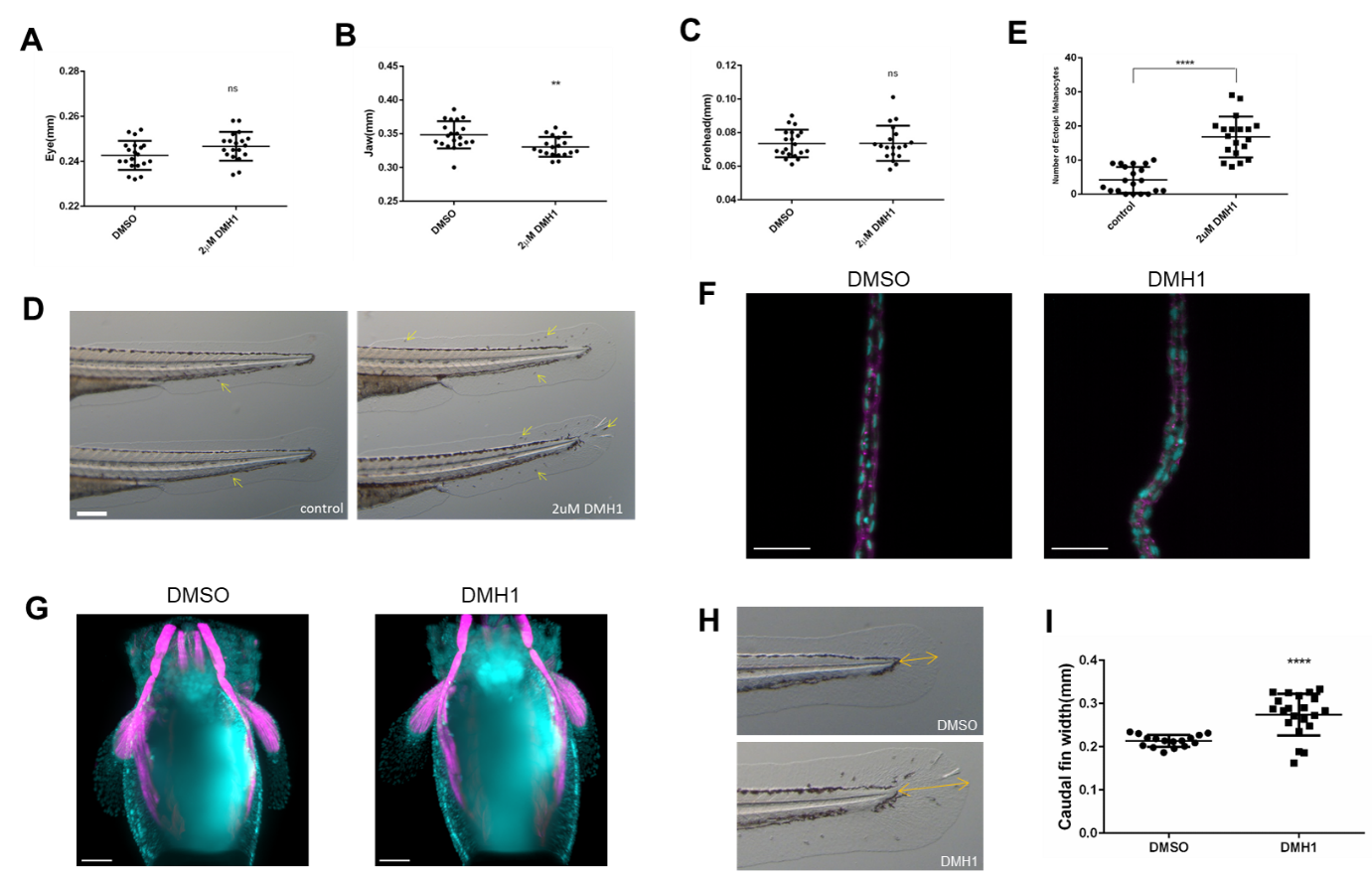


**Supplementary Figure 2: Inhibition of Bmp signaling inhibition does not lead to pronounced changes in the gross morphology of zebrafish embryo**

(**A**) Size of the eyes was measured in 76hpf DMSO- or DMH1-treated embryos. (**B**) Size of the jaw was measured in 76hpf DMSO- or DMH1-treated embryos. (**C**) Size of the forehead was measured in 76hpf DMSO- or DMH1-treated embryos. (**D**) The number of melanophores was assessed in 76hpf DMSO- or DMH1-treated embryos. Arrows point melanophores within the ventral fin fold. Scale bar: 50μm. (**E**) Quantification of the number of melanophores in DMSO- or DMH1-treated embryos (n=20). (**F**) Optical section showing the mediolateral axis of the ventral fin fold in 76hpf DMSO- or DMH1-treated embryos (n=11). Scale bar: 50μm. (**G**) Optical section showing the pectoral fins of 76hpf DMSO- and DMH1- treated embryos from the ventral side (n=11). (**H**) The caudal fin of 76hpf DMSO- or DMH1-treated embryos. Attenuation of Bmp signaling significantly increased the expansion of the caudal fin along the anteroposterior axis. (**I**) Quantification of the caudal fin outgrowth in DMSO- or DMH1-treated embryos (n=20). Scale bar: 100μm. **p*<0.05, ***p*<0.01, ****p*<0.001, *****p*<0.0001. ns: not significant.


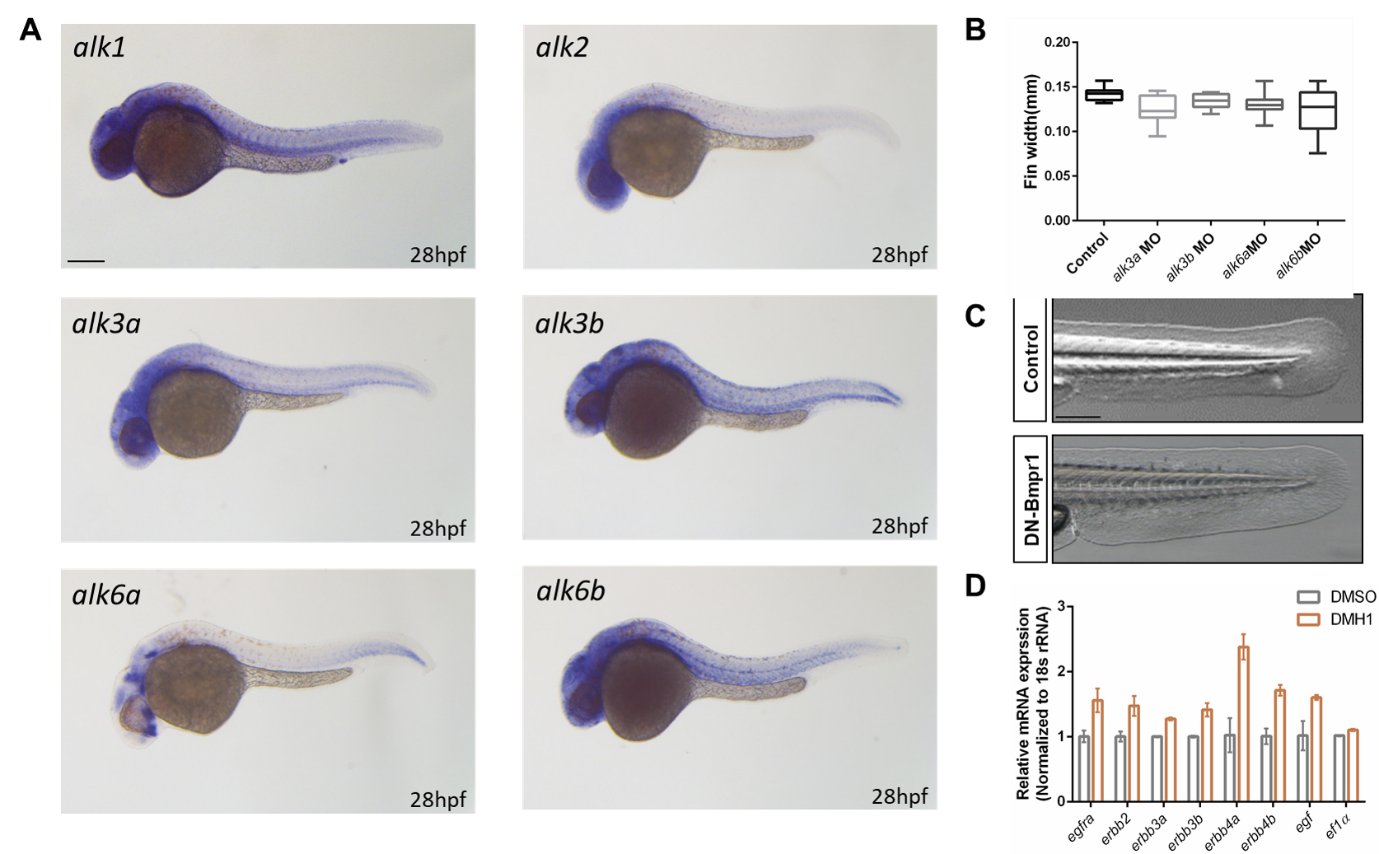


**Supplementary Figure 3: Bmpr1s function in a redundant and overlapping manner to mediate Bmp signaling in the ventral fin fold**

(**A**) Expression of Bmpr1s was analyzed at 28hpf by *in situ* hybridization. (**B**) The proximodistal extension of the ventral fin fold in control and MO-injected embryos was quantified. Injection of MO targeting individual *Bmpr1* did not have any discernible effects on the proximodistal expansion of the ventral fin fold (n=25 per MO) (**C**) Ectopic expression of DN-Bmpr1 recapitulates the fin phenotype in DMH1-treated embryos. (**D**) Expression of Egf signaling components, in particular, receptors measured by quantitative RT-PCR appeared to be drastically increased in the absence of Bmp signaling.


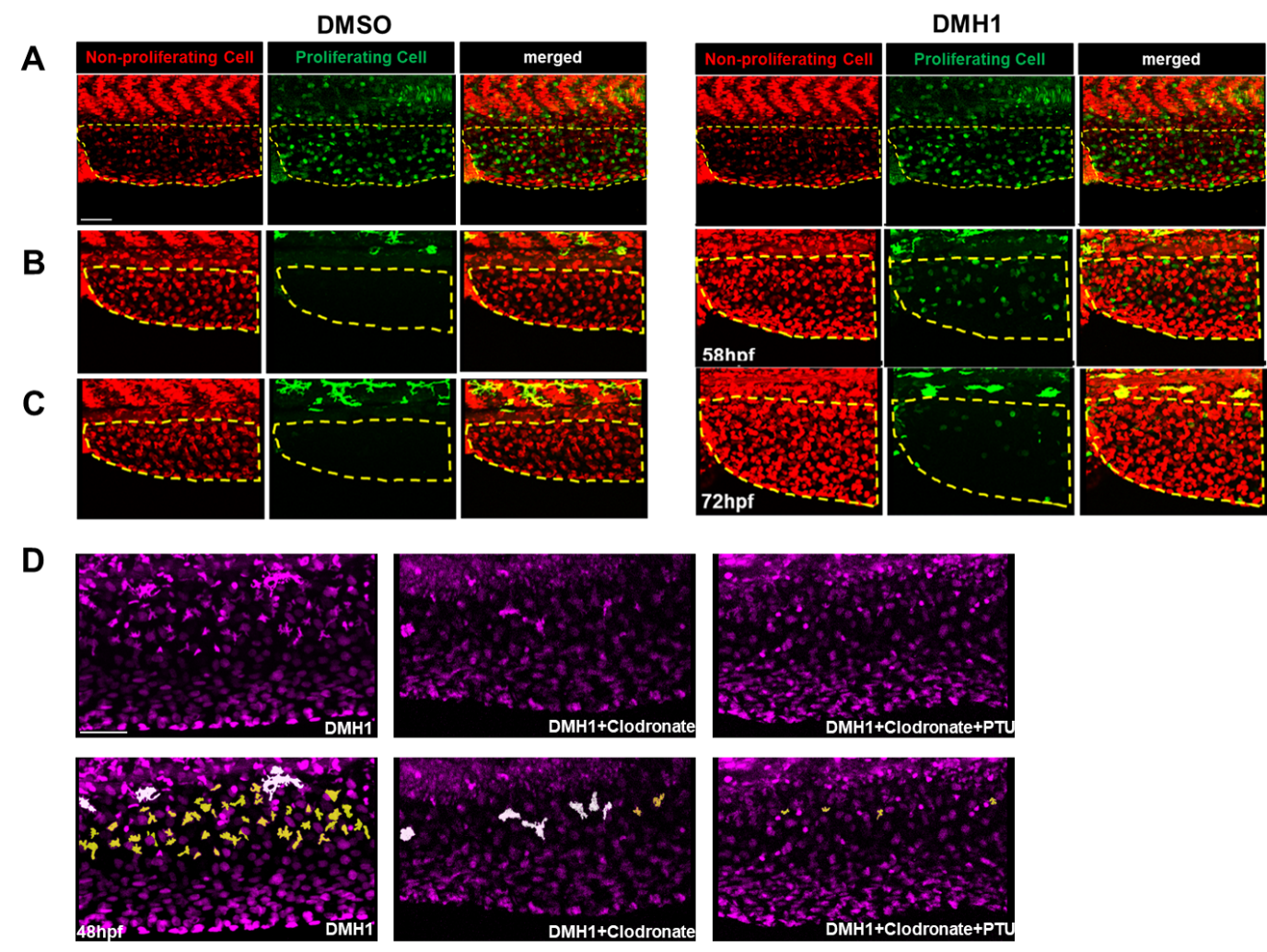


**Supplementary Figure 4: The proliferative capacity of the ventral fin fold is sustained upon Bmp signaling inhibition**

(**A**-**C**) The numbers of Cerulean positive proliferating cells and mCherry positive non-proliferating cells were analyzed in DMSO- or DMH1-treated embryos at 28hpf (**A**), 58hpf (**B**), and 72hpf (**C**). (**D**) The ventral fin fold of 76hpf DMH1- (left), DMH1/clodronate- (middle), or DMH1/clodronate/PTU- (right) treated embryos. Treatment with clodronate and PTU, which respectively target immune cells and melanocytes, largely abrogated the cells with non-epithelial morphology, which were excluded from further analyses. Arrows and arrowheads point immune cells and melanocytes respectively. Scale bar: 50μm


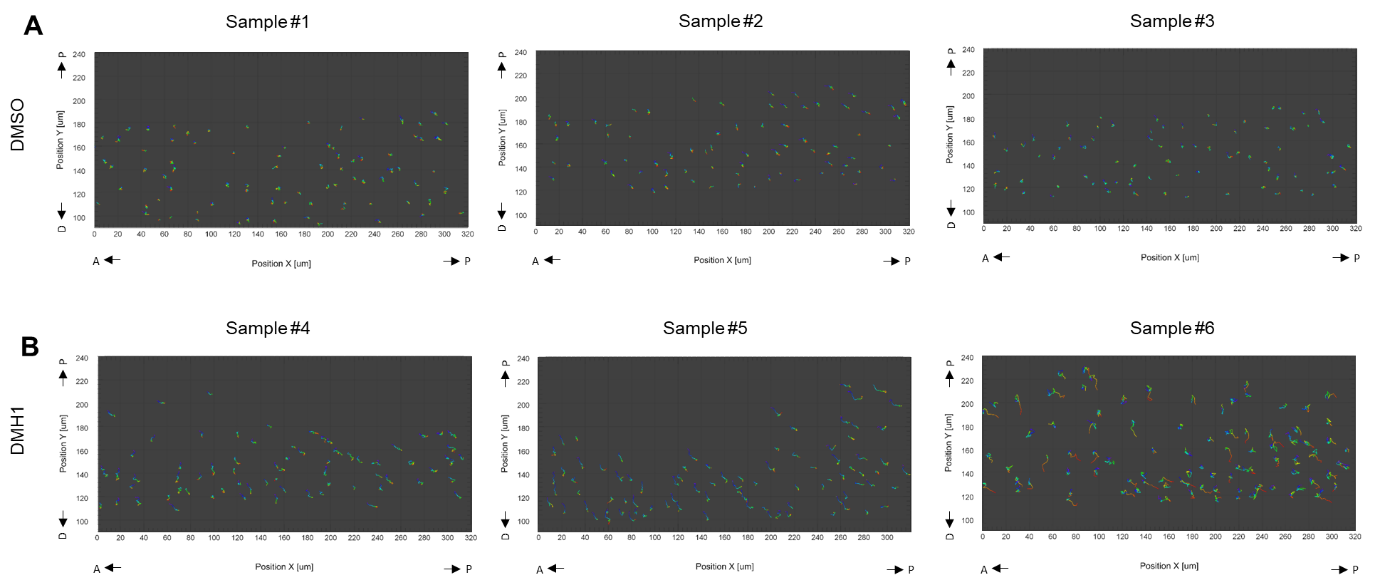


**Supplementary Figure 5: Inhibition of Bmp signaling promotes navigating behaviors of cells within the ventral fin fold**

The migratory tract of individual cells within the ventral fin fold was assessed in DMSO-treated embryos (**A**) or DMH1-treated embryos (**B**).


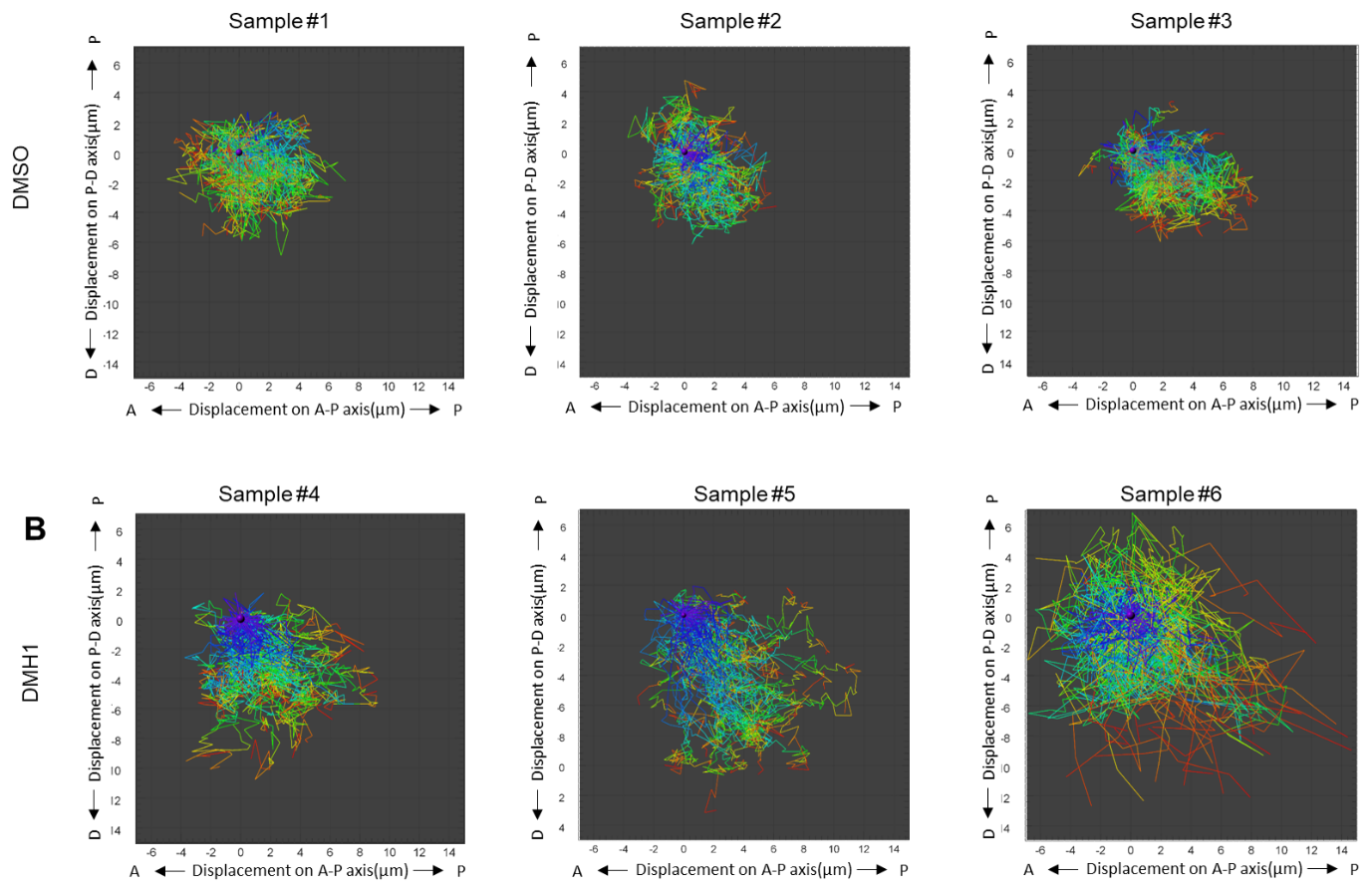


**Supplementary Figure 6: Inhibition of Bmp signaling promotes distal migration of cells within the ventral fin fold**

The composite migratory tract of individual cells within the ventral fin fold was assessed in DMSO-treated embryos (**A**) or DMH1-treated embryos (**B**). X and Y axes represent the anteroposterior and proximodistal axes respectively. The position of individual cells at 40hpf is given the value of (0,0).

**Supplementary Table**

**
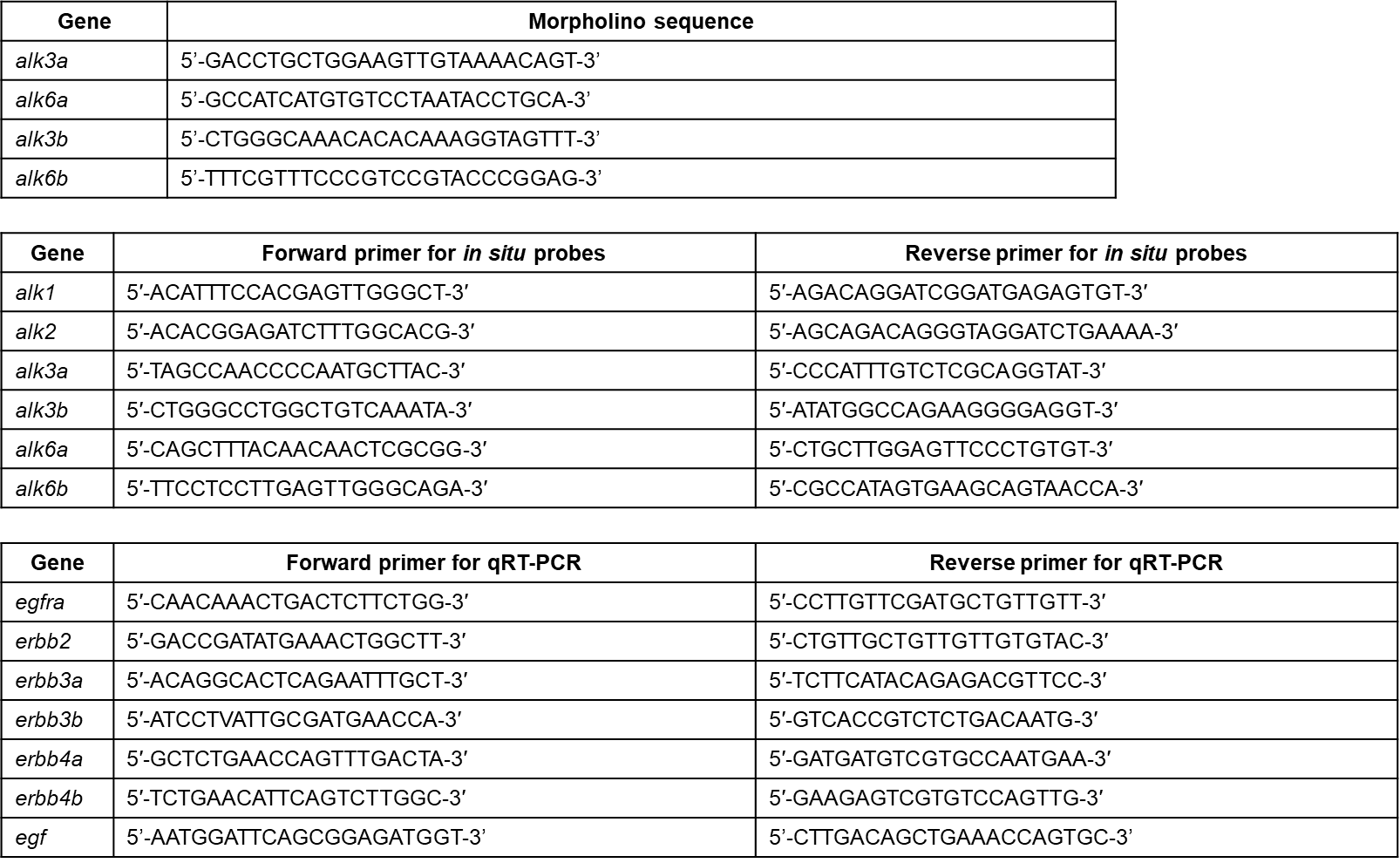
**

**Supplementary Table 1: List of morpholinos and primers used**

**Supplementary Movies**

**Supplementary Movie 1: 3D rotation of the embryos treated with DMSO or DMH1 in the area surrounding the pectoral fins**

**Supplementary Movie 2: Time lapse imaging of DMSO-treated embryos from 48hpf to 60hpf**

**Supplementary Movie 3: Time lapse imaging of DMH1-treated embryos from 48hpf to 60hpf**
